# Mechanical Behavior Optimization of Chitosan Extracted from Shrimp Shells as a Sustainable Material for Shopping Bags

**DOI:** 10.3390/jfb9020037

**Published:** 2018-05-22

**Authors:** Giacomo D’Angelo, Amal Elhussieny, Marwa Faisal, I. S. Fahim, Nicola M. Everitt

**Affiliations:** 1 Bioengineering Research Group, Faculty of Engineering, University of Nottingham, University Park, Nottingham NG7 2RD, UK; Giacomo.D’Angelo@nottingham.ac.uk; 2 Centre of Nano-science and technology (CNT), The Nile University, Nile Avenue, Giza 116453, Egypt; am.abdullah@nu.edu.eg (A.E.); m.faisal@nu.edu.eg (M.F.); 3 Department of Industrial Engineering, School of Engineering, The Nile University, Nile Avenue, Giza 116453, Egypt; 4 Department of Mechanical Materials and Manufacturing Engineering, University of Nottingham, Nottingham NG7 2RD, UK; Nicola.Everitt@nottingham.ac.uk

**Keywords:** biocomposites, chitosan, tensile strength

## Abstract

The use of biodegradable materials for shopping bag production, and other products made from plastics, has recently been an object of intense research—with the aim of reducing the environmental burdens given by conventional materials. Chitosan is a potential material because of its biocompatibility, degradability, and non-toxicity. It is a semi-natural biopolymeric material produced by the deacetylation of chitin, which is the second most abundant natural biopolymer (after cellulose). Chitin is found in the exoskeleton of insects, marine crustaceans, and the cell walls of certain fungi and algae. The raw materials most abundantly available are the shells of crab, shrimp, and prawn. Hence, in this study chitosan was selected as one of the main components of biodegradable materials used for shopping bag production. Firstly, chitin was extracted from shrimp shell waste and then converted to chitosan. The chitosan was next ground to a powder. Although, currently, polyethylene bags are prepared by blown extrusion, in this preliminary research the chitosan powder was dissolved in a solvent and the films were cast. Composite films with several fillers were used as a reinforcement at different dosages to optimize mechanical properties, which have been assessed using tensile tests. These results were compared with those of conventional polyethylene bags used in Egypt. Overall, the chitosan films were found to have a lower ductility but appeared to be strong enough to fulfill shopping bag functions. The addition of fillers, such as chitin whiskers and rice straw, enhanced the mechanical properties of chitosan films, while the addition of chitin worsened overall mechanical behavior.

## 1. Introduction

Over the last decades, the world has experienced a greater need for synthetic plastics. Due to the high usage and mismanagement of plastics, pollution has emerged everywhere. For more than 50 years, the consumption and global production of plastics have been increasing [1]. In 2003, 299 million tons of plastics were produced, increasing by four percent in 2012. In 2008, an estimated 260 million tons of plastics were produced, and at the end of 2015, the consumption rate reached 297.5 million tons [1]. Plastics are used in many products due to their attractive qualities, such as being lightweight, flexible, strong, low cost, and having a high moisture resistance. However, due to their slow degradation, there is a huge waste problem. A study conducted by a scientific working group at UC Santa Barbara’s National Centre for Ecological Analysis and Synthesis (NCEAS), determined the input of plastic waste from land into oceans. Jambeck et al. [1] reported that eight million metric tons of plastics end up in the oceans every year, which is equal to five grocery bags filled with plastic for every foot of coastline in the world. In this environment, plastics can cause numerous difficulties for organisms that often mistake them for food and suffocate, or become entangled in the bags and drown. Furthermore, their annual input into oceans will double in 2025, reaching nearly 20 times the amount of eight million metric tons.

One of the greatest forms of plastic pollution is plastic shopping bags. In Egypt, two percent of the Municipal Solid Waste ends up in landfills; 2% is recycled, and 8% is composted, while 88% is disposed of in open areas. The plastic pollution in Egypt occurs as it is not naturally decomposable [2]. The remainder accumulates in the street or at illegal dumping sites, generating environmental and public health problems. Natural biopolymers are an environmentally friendly substitute for plastic packaging if performance, processing and cost can be controlled. The use of a local food waste shrimp shells as the source for biodegradable bags, turns the shells from being part of the food waste problem to being part of the solution.

Chitosan is produced by the deacetylation of chitin, which is the main substance in the exoskeleton of crustaceans. Chitosan is a linear polysaccharide of 2-amino-2-deoxy-β-d-glucopyranose units linked by (1-4)-β-glucosidic linkage. The advantages of using chitosan in plastics are its biocompatibility in vivo, controllable degradation rate, and enhanced mechanical properties, toughness, and transparency [3]. The mechanical strength of chitosan varies with the degree of deacetylation, different types of chitosan, plasticizer concentrations and solvent type [4] and it can reach values comparable with those of commercial polypropylene (PP) and polyethylene (PE) [5,6]. However, chitosan is less flexible, stretchable than the plastic films [7]. This study aimed to enhance the mechanical strength of pure chitosan using different extraction techniques for the chitosan matrix, different dosages and types of fillers blended with chitosan for film manufacturing [8]. Cellulose, nano-cellulose extracted from rice straw, chitin, and chitin whiskers were used as fillers, because they are natural polysaccharides and widely used in different applications due to their excellent mechanical strength [9,10].

## 2. Materials and Methods

### 2.1. Materials

#### 2.1.1. Preparation of Chitosan

Chitosan was extracted from shrimp shells waste with two different methods: (i) in the first method, chitosan was extracted from the shrimp shells waste ground with an electric grinder; (ii) in the second method chitosan was extracted from the entire exoskeleton of shrimp shell waste complete with head and tail—without grinding. This latter method has shown better thermal and physiochemical properties than from the grounded form [11]. The following procedures were applied for both (i) and (ii), starting with a deproteinization step, followed by a demineralization step to produce chitin, to be used later as a filler. The final step was deacetylation using 50% NaOH to produce chitosan [11].

#### 2.1.2. Preparation of Fillers

Four types of fillers were used: Cellulose, nano-cellulose, chitin and chitin whiskers. The cellulose was extracted from rice straw waste with chemical treatment to remove the lignin and undesired materials by using Alkali treatment [8]. The cellulose produced was sonicated by using an Ultrasonicator for 30 min [8], while chitin and chitin whiskers were extracted from shrimp shell waste with chemical treatment by a demineralization process using 3 N of HCl [8].

#### 2.1.3. Preparation of Composites

**Figure 1:**
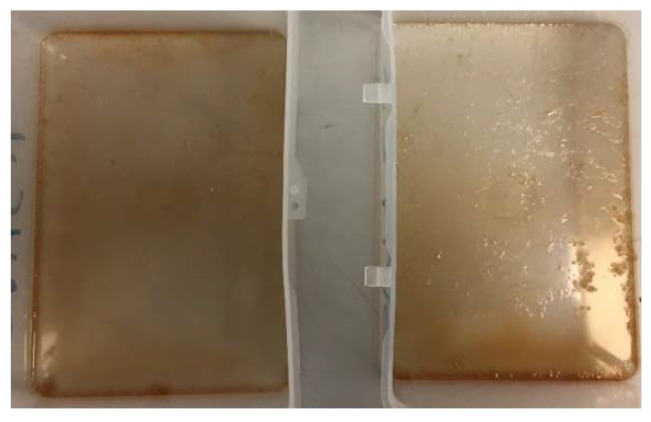
Chitosan composite drying at room temperature.

Different composites of chitosan and filler were manufactured to optimize chitosan mechanical behavior. Each sample consisted of a matrix of chitosan and a filler in different dosages (as reported Table 1), for a total weight of 1 g. All composites were dissolved in 1% CH_3_COOH, at 150 °C. Finally, the solutions were poured into flat flexible plastic covers, and then left to dry at room temperature [8,11] (Figure 1). Chitosan composites were not neutralized for this laboratory study.

**Figure 2:**
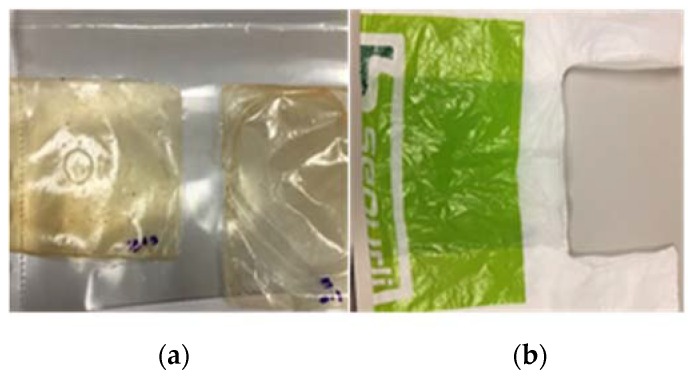
The visual appearance of (**a**) rectangular chitosan samples and (**b**) conventional plastic bags.

The thin films obtained had thicknesses ranging from 73 to 197 µm and a rectangular shape, as shown in Figure 2a. 

#### 2.1.4. Egyptian Plastic Bags

To assess the suitability of these materials as biodegradable shopping bags in terms of tensile mechanical behavior, their performances were compared with those of conventional polyethylene bags used in Egypt (Figure 2b.) Specimens obtained from these bags had an average thickness of approximately 28 µm.

### 2.2. Methods

**Figure 3:**
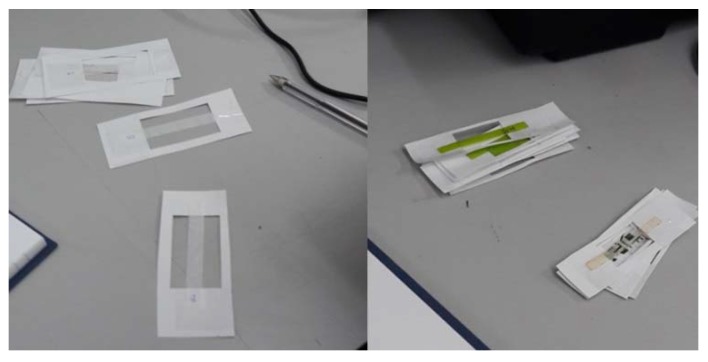
Plastic bags and chitosan specimens mounted on the paper frame before the tensile test.

With the aim of assessing mechanical performance of conventional plastic bags and chitosan specimens, tensile tests were carried out using the following testing protocol, based upon European standards for the determination of tensile properties for plastics [12,13,14,15]. Samples were cut in thin rectangular strips, 50 mm by 5 mm (Figure 3), leaving a distance between grips of 26 ± 2 mm for the tensile test. These dimensions, which scaled those recommended by standards, by a factor of 2 [12] represented an optimum trade-off to test a statistically meaningful number of specimens for each chitosan sample (due to the limited amount of material), especially when considering the comparative nature of this experimental investigation. Specimens were cut by using laser equipment, which ensured better repeatability, precision and effectiveness in comparison to other methods considered (extrusion by stamp). In the case of Egyptian plastic bags, to take into account the anisotropy of the material (due to the manufacturing process), specimens were cut in both vertical (V—parallel to the handle) and horizontal (H—perpendicular to the handle) directions. According to standards [14], before and after the cutting process, materials were kept at normal room temperature and humidity levels (23 ± 2 °C and 50 ± 10% R.H.). After cutting, the thickness of each strip specimen was measured using a micrometer. Ten measurements along the full length were undertaken for each strip, using the average as the nominal parameter for the tensile test. Thereafter, each strip was numbered and stuck using cello tape on a paper mount, as shown in Figure 3. The sides of the mount were cut once the sample was positioned in the tensometer.

**Figure 4:**
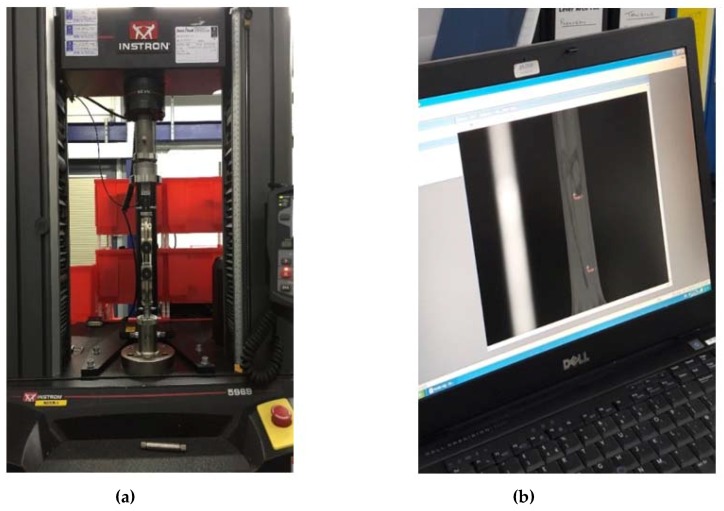
(**a**) Instron tensile test equipment and (**b**) extension measurement with optical extensometers.

This procedure provided a rigid frame for each of the specimens—allowing for precise vertical positioning of the specimen while preventing its slippage during the test—because of greater friction between the sample material and the metal grips. Ultimately, this method facilitated measuring the extension of specimens by allowing setting of the original distance between grips within the range established. Two relevant methods were considered: (i) optical extensometers (more precise but suitable only for tracking small deformation (Figure 4) and (ii) extension recorded from the load cell crosshead. For the first method, specimens needed to be painted to allow the optical extensometer to track selected targets. This procedure could have altered the measured chitosan properties (due to solvent effects from the paint). The optical method is also less suitable for rough surfaces (as those of chitosan cast samples). Therefore, it was initially applied only to a few plastic bag specimens to compare the effectiveness of both measurements before extending the procedure to all of the specimens.

**Figure 5:**
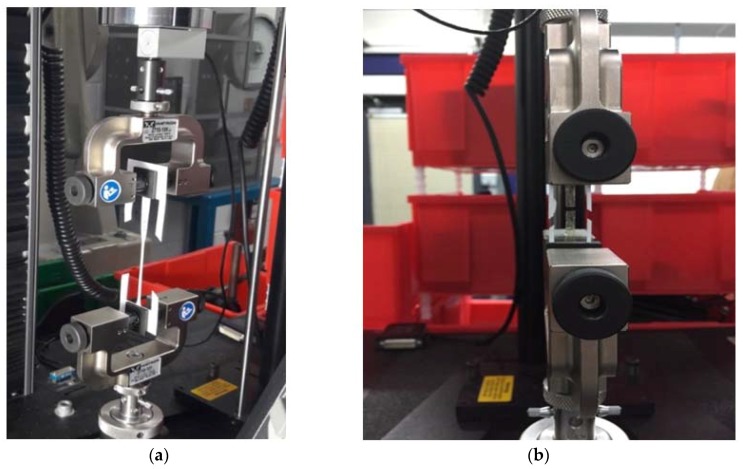
The visual appearance of tensile test running for (**a**) conventional plastic bags and (**b**) chitosan specimen after fracture.

Tensile tests were carried out using Instron table top load cells with 50 kN maximum load. Each specimen was placed vertically between the grips with the help of the paper frame, which was cut afterwards, to act as a gripping enhancer (Figure 5). After the application of a pre-load (with stress not exceeding Young modulus/2000) [14], specimens were loaded in displacement control mode (5 mm/min) until fracture.

Load and displacement were recorded during the test through the load cell and crosshead measurements with a data acquisition frequency of 10 Hz. Raw data analysis allowed the calculation of yield strength, fracture strength, Young’s modulus and strain at break (indicating the ductility of the material), used for the mechanical characterization of each material. At least five tests were carried out for each material and results were reported as average values with relative standard errors [14]. 

## 3. Results

**Figure 6:**
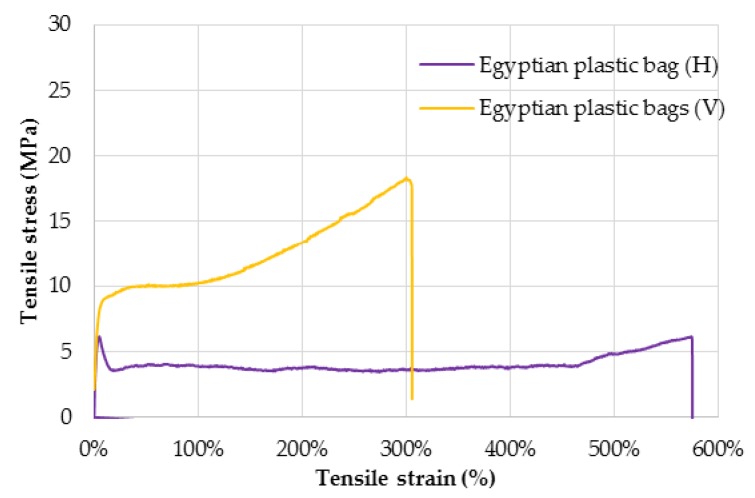
Examples of stress versus strain trend for Egyptian plastic bag specimens for both horizontal (H) and vertical (V) orientations. n = 2.

Results from tensile tests are typically analyzed in a stress versus strain plot—useful to calculate the most relevant parameters mentioned above, in accordance with standards [14]. For instance, Figure 6 shows the typical curves exhibited by Egyptian plastic bag specimens cut in both horizontal and vertical directions. For this specific material, a different behavior between H and V specimens can be seen because of the anisotropy due to the manufacturing process. In the following sections, this observation is discussed.

### 3.1. Comparison of Extension Measurement Methods for Young Modulus Evaluation

Measuring a stiffness modulus using the crosshead movement is potentially less accurate than if a direct measurement of extension can be obtained. Since other considerations meant that the crosshead data was the only extension data available to us for the chitosan specimens, the modulus evaluation method had to be validated. For this purpose, for tests reported in Table 2, two methods for measuring the extension were used as mentioned above: (i) optical extensometers and (ii) extension recorded from the load cell crosshead. Consequently, Young’s moduli evaluated from these measurements were compared, as reported in Table 2.

There was no significant difference between moduli calculated with the two methods, (ANOVA). For the main tests reported below, only the crosshead method was used to avoid any possible influence of paint on chitosan properties.

### 3.2. Chitosan Biodegradable Bags vs. Conventional Plastic bags

Table 3 summarizes tensile mechanical properties (mean values and standard errors) of Egyptian plastic bag specimens, for both cut orientations, compared with those of pure chitosan to assess the suitability of this material, to be employed for biodegradable shopping bags.

Firstly, the anisotropic mechanical behavior of plastic bags, also seen in Figure 6, is highlighted. Differences with a factor of two can be observed, in the fracture strength and the strain at break, indicating a more ductile behavior in the horizontal direction and a higher resistance in the vertical direction. However, yield strengths and moduli are comparable for both directions.

In reference to conventional plastic bags, chitosan exhibited a sensibly higher modulus and a similar tensile resistance while ductility (related to the fracture strain) was significantly lower. This observation indicates that this biodegradable material could be suitable to withstand mechanical stresses, but perhaps not ductile enough for the proposed use (at least when manufactured by the casting method).

Different combinations of chitosan and fillers, varying the type and the dosage were tested to optimize these mechanical properties of pure chitosan.

### 3.3. Optimization of Chitosan Composite Materials

**Figure 7:**
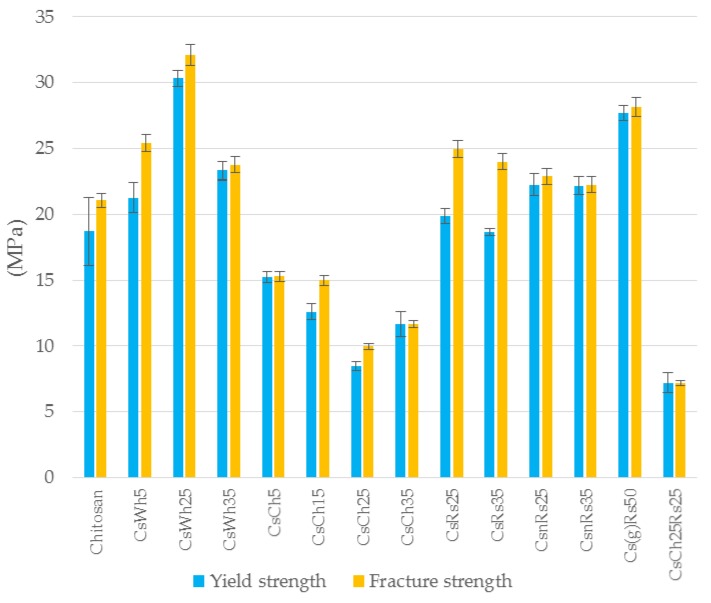
Yield strength and fracture strength from the tensile test for all chitosan specimen combinations (sample IDs from Table 1. n = 2).

**Figure 8:**
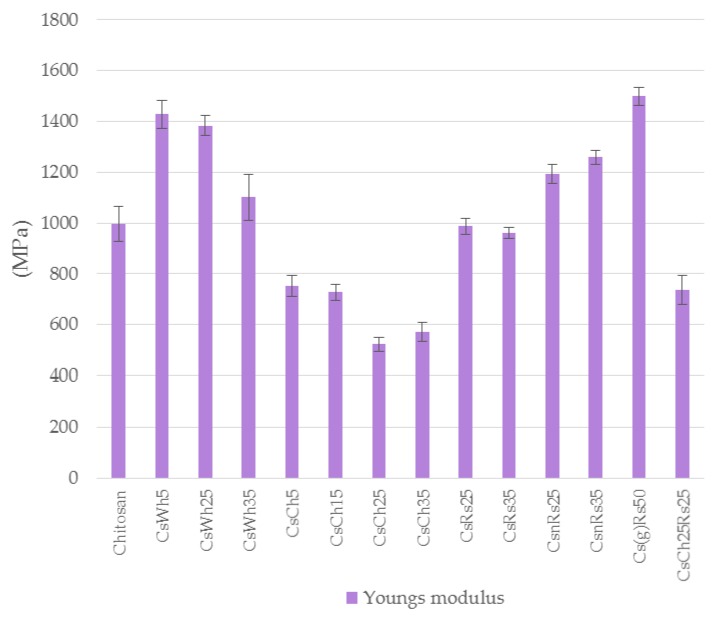
Young modulus from the tensile test for all chitosan specimen combinations (sample IDs from Table 1), n = 2.

**Figure 9:**
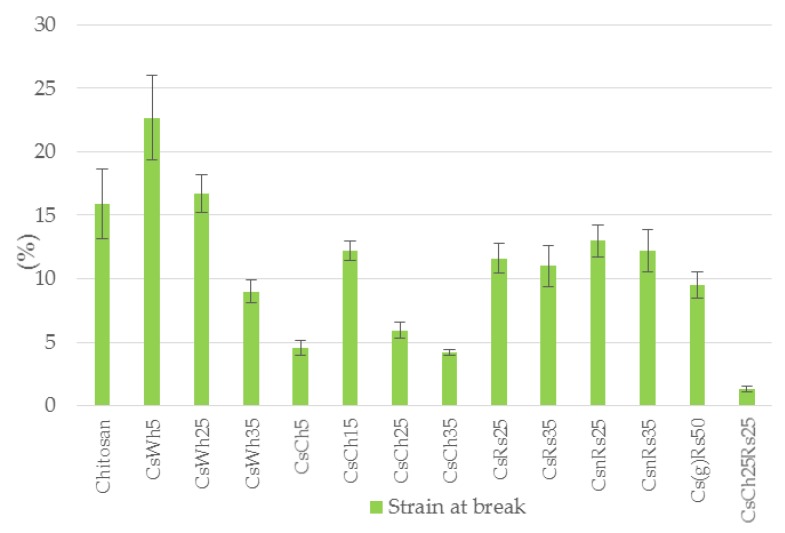
Strain at break from the tensile test for all chitosan specimen combinations (sample IDs from Table 1), n = 2.

Figure 7, Figure 8 and Figure 9 show results concerning yield and fracture strength, Young’s modulus and strain at break, respectively, for all chitosan combinations analyzed in the present study.

Figure 7 shows that all combinations containing chitin exhibited worse tensile strengths than pure chitosan. The strengths decreased with an increasing amount of chitin. The other fillers instead had overall, a positive effect. In particular, rice straws with chitosan specimens (CsRs25 and CsRs35), which presented yield strengths similar to pure chitosan, exhibited the highest hardening (increase in the fracture strength) within the combinations analyzed. Different behavior was observed for nano Rice straw specimens with the same dosages, which had almost constant values of yield and fracture strength (higher than pure chitosan). Ground chitosan with the highest dosage of rice straws (Cs(g)Rs50) presented the highest strength values among rice straw combinations. In this regard, further tests are needed to understand if these results are due to the matrix or the filler dosage. With the whiskers, a significant improvement in tensile performance with respect to chitosan was noted, especially for 25% dosage (CsWh25), which appears to be the optimum for this type of filler. A non-negligible hardening at lower dosages was observed.

Looking at the Young’s moduli presented in Figure 8, again, all chitin combinations presented worse performance than pure chitosan, ground chitosan with rice straws exhibiting the highest value of stiffness. Whiskers combinations increased the performance of pure chitosan, in lower extent with increasing dosages.

Considering strain to failure (Figure 9), the same trend is observed, where for dosage higher than 25% whiskers worsened pure chitosan performance. This property decreased overall with increasing dosages of filler (except for low dosages of chitin), the best example being CsCh25Rs25, where 50% filler used drastically decreased pure chitosan value. This decrease indicates that pure chitosan’s structure allows for a plastic extension—hardly improvable with these types of fillers. Note by comparing results amongst each filler category, only in the case of rice straw specimens, low ductility corresponded to high fracture strength and modulus (e.g., Cs(g)Rs50), as would be expected. In the case of the other fillers, no clear correlations could be established between mechanical parameters. This outcome slightly complicated the optimization process. However, when combining all the tensile mechanical parameters, it may be concluded that the optimum filler seems to be whiskers in dosages from 5% to 25%, which can provide higher yield and fracture strength together with Young’s modulus, while not decreasing pure chitosan ductility properties.

## 4. Conclusions

The present study assessed the feasibility of chitosan as biodegradable material for shopping bags. Firstly, conventional polyethylene bags used in Egypt were tested as reference materials, allowing definition and testing protocol for the following tests. Then, several combinations of chitosan and fillers in different dosage identified the optimum mix of strength, modulus, and ductility. Results obtained led to the following conclusions:

- No significant differences were observed between the optical and crosshead extension measurement used for plastic bag specimens (ANOVA). This observation validated the latter method, which was used for tests on chitosan.
- Plastic bags presented isotropic behavior, being vertical specimens more resistant (double fracture strength) and less ductile (half strain at break) than horizontal specimens.
- Results obtained for pure chitosan indicated that this biodegradable material could withstand mechanical stresses but perhaps, at least with casting manufacturing, would not be ductile enough for the proposed use. This last property was not enhanced by the majority of fillers used, especially by chitin which overall, worsened all the mechanical performances.
- Rice straw specimens presented lower ductility than pure chitosan. However, this filler enhanced both resistance and modulus of pure chitosan especially in the case of ground chitosan with the highest rice straw dosage. Further studies are needed to understand whether this enhancement was due to the matrix or the filler dosage.
- Whiskers appeared to have an overall positive effect in all tensile properties analyzed, being dosages from 5% to 25%, an optimum range for improving yield and fracture strength together with Young’s modulus, while not decreasing, or even enhancing pure chitosan ductility properties.

## Figures and Tables

**Table 1:** Chitosan specimens tested: When unspecified chitosan was extracted from the whole shells (method ii) (only for one sample), Cs (g) Rs50, chitosan was extracted from ground shells (method i); the dosage (in % by weight of the total weight) of each component is indicated in brackets.

Sample	Specimen ID
Chitosan	Chitosan
Chitosan + chitin Whiskers (5%)	CsWh5
Chitosan + chitin Whiskers (25%)	CsWh25
Chitosan + chitin Whiskers (35%)	CsWh35
Chitosan + Chitin (5%)	CsCh5
Chitosan + Chitin (15%)	CsCh15
Chitosan + Chitin (25%)	CsCh25
Chitosan + Chitin (35%)	CsCh35
Chitosan + Rice straw (25%)	CsRs25
Chitosan + Rice straw (35%)	CsRs35
Chitosan + nano Rice straw (25%)	CsnRs25
Chitosan + nano Rice straw (35%)	CsnRs35
Chitosan (ground shells) + Rice straw (50%)	Cs(g)Rs50
Chitosan + Chitin (25%) + Rice straw (25%)	CsCh25Rs25

**Table 2:** Comparison between crosshead and optical extensometer in measuring Young modulus carried out for some Egyptian plastic bag specimens (H). n = 2.

Specimen ID	Young Modulus (MPa)	
	Optical Extensometer	Crosshead
EH1	176	192
EH2	201	199
EH3	199	198
Mean	197	191
Standard error	2.05	6.39
ANOVA, *p*-value = 0.562		

**Table 3:** Comparison between pure chitosan and Egyptian plastic bags for tensile mechanical properties: Mean values and standard errors. N = 2.

Material Tested	Yield Strength	Fracture Strength	Young Modulus	Strain at Break
	(MPa)	(MPa)	(MPa)	(%)
EV	10.39 ± 0.79	22.06 ± 1.59	179.4 ± 4.2	304.70 ± 17.88
EH	9.84 ± 0.65	11.01 ± 1.16	205.9 ± 5.1	617.92 ± 50.66
Chitosan	18.73 ± 2.57	21.08 ± 2.16	995.3 ± 68.5	15.93 ± 2.73
